# Gemcitabine–oxaliplatin as a bridge therapy toward autologous hematopoietic stem cell transplantation in infant-type brain tumors

**DOI:** 10.3389/fonc.2025.1476411

**Published:** 2025-05-13

**Authors:** Barbara Castelli, Carla Fonte, Marco Tellini, Marco Di Nicola, Milena Guidi, Laura Giunti, Bianca Tirinnanzi, Chiara Marzano, Anna Maria Buccoliero, Ludovico D’Incerti, Flavio Giordano, Mirko Scagnet, Veronica Tintori, Lorenzo Genitori, Iacopo Sardi

**Affiliations:** ^1^ Neuro-Oncology Unit, Meyer Children’s Hospital IRCCS, Florence, Italy; ^2^ Pathology Unit, Meyer Children’s Hospital IRCCS, Florence, Italy; ^3^ Radiology Unit, Meyer Children’s Hospital IRCCS, Florence, Italy; ^4^ Neurosurgery Unit, Meyer Children’s Hospital IRCCS, Florence, Italy; ^5^ Pediatric Hematology/Oncology and HSCT Department, Meyer Children’s Hospital IRCCS, Florence, Italy

**Keywords:** brain tumors, gemcitabine, oxaliplatin, high-dose chemotherapy, autologous stem cell transplantation, pediatric oncology

## Abstract

**Introduction:**

In neuro-oncological pediatric patients under 3 years of age, chemotherapy intensified to high doses (high-dose chemotherapy, HDC) represents the cornerstone to avoid the potential toxicity of radiotherapy. Combination treatment with gemcitabine–oxaliplatin (GemOx) was administered for infant- type cerebral tumors as a bridge toward autologous hematopoietic transplantation to achieve clinical and neuroradiological permissiveness to HDC and to raise the possibility of second-look neurosurgery.

**Methods:**

From May 2017 to May 2023, at Meyer Children’s Hospital IRCSS in Florence (Italy), four patients, with a median age of 19 months (with two high- grade gliomas, a metastatic medulloblastoma, and a choroid plexus carcinoma CNS WHO grade 3), were subjected to partial neurosurgical removal and induction therapy delivered according to the Italian program for malignant cerebral tumors under 3 years. To delay HDC, either for disease reassessment or for temporary unfitness, GemOx cycles were administered. Gemcitabine 1,000 mg/m2 and oxaliplatin 100 mg/m2 were given on day 1 every 21–28 days for one to six cycles.

**Results:**

The treatment was well tolerated overall, except for severe platelet hematological toxicity in a patient, which required dose reduction to 75%. After GemOx, one patient was also subjected to further neurosurgery. Bridge therapy made it possible to submit patients to HDC in safety, in permissive clinical conditions, and after assessment of disease stability.

**Conclusion:**

In infant-type cerebral tumors eligible for HDC, GemOx could be a possible strategy in the case of post-induction residual disease to exclude uncertain evolution or when waiting for clinical suitability for second surgery and intensified treatment. The therapy was overall safe and well tolerated. This approach resulted incisive in the therapeutic or palliative choice for extremely young patients with aggressive brain tumors.

## 1 Introduction

Gemcitabine (Gem) and oxaliplatin (Ox) are both active drugs for a large variety of solid tumors, with no overlapping toxicity (1). Gemcitabine (2′,2′-difluoro-2′-deoxycytidine) is a synthetic deoxycytidine that exhibits its cytotoxic effects primarily through the inhibition of DNA synthesis (2). It remains a cornerstone in locally advanced or metastatic pancreatic cancer (2). Oxaliplatin, a platinum-based chemotherapeutic drug, has been shown to play a definite role in the management of colorectal cancer, representing a new standard of treatment in the adjuvant setting and for metastatic diseases (3). Oxaliplatin is a diaminocyclohexane (DACH)-platinum compound that is active through the formation of DNA adducts, which are not recognized by mismatch repair complexes (4). The absence of nephrotoxicity, its lack of need for intravenous hydration, and its lower gastrointestinal and hematological toxicity rates are the main advantages of oxaliplatin over cisplatin (1). Numerous clinical studies have evaluated the combination of gemcitabine–oxaliplatin (GemOx) in adult solid tumors, including a phase III trial in pancreatic cancer (4) and a phase II study in advanced biliary tract carcinomas (5).

The pharmacokinetics of GemOx do not differ significantly in children compared with those in adults (4). In 2011, Geoerger et al. performed a multicenter, non-randomized phase II study to assess the objective response rates after four cycles of gemcitabine in combination with oxaliplatin in children and adolescents with relapsed or refractory solid tumors, inclusive of medulloblastoma and other central nervous system (CNS) tumors (4). The GemOx combination administered on a biweekly schedule showed an acceptable safety profile, but limited activity, in this pediatric cohort (4). A further improved tumor control was observed with prolonged treatment, particularly in a case of medulloblastoma and a papillary tumor of the pineal region (4).

Moreover, Bender et al., in a retrospective, single-center study, reported on a single-institution experience combining anti-CD20 therapy with GemOx for mature B-cell non-Hodgkin’s lymphoma (NHL) in children and adolescents who were unfit for intensive chemotherapy due to significant comorbidities (6). Indeed, for patients who cannot tolerate intensive regimens due to underlying comorbidities, the optimal treatment strategy remains unknown (6).

In children younger than 3 years with brain tumors, the main therapeutic approaches are surgery and chemotherapy to delay radiotherapy: indeed, the exposure of immature CNS to radiotherapy can induce early and severe cognitive deficits (7). In our institution, the preferred chemotherapy approach is delivered according to the Italian program for malignant CNS tumors under 3 years schedule (8). The induction phase consists of four courses and it includes the following drugs: methotrexate, vincristine, etoposide, cyclophosphamide, carboplatin. Peripheral blood stem cells are collected for rescue therapy after the second course. The intensification and consolidation phases include two high-dose chemotherapy (HDC) regimens (7). In 2020, our group evaluated the safety and the effectiveness of high-dose thiotepa and carboplatin/thiotepa followed by stem cell rescue in congenital brain tumors, which showed a high response rate (7).

However, to date, there are limited data on the treatment of infant patients who are clinically not suitable to receive intensified treatments, either temporarily due to reversible comorbidities or due to the need for further disease response assessment. Therefore, in our cohort of these patients, GemOx therapy was administered as a bridge toward autologous hematopoietic transplantation in the event of uncertain response to the induction therapy needing evolution re-evaluation, while waiting for clinical permissiveness for more intensive treatments, or when considering the possibility of a second-look neurosurgery before intensified regimens.

## 2 Patients and methods

This retrospective, single-center study was approved by the Ethical Committee and was performed in accordance with the principles of the Declaration of Helsinki. A total of four patients (two male and two female children; median age, 19 months; age range, 8–28 months) affected by infant-type brain tumors and who were treated at the Neuro Oncology Unit in Meyer Children’s Hospital IRCCS in Florence, Italy, were included. The included patients presented the following: a high-grade glioma (HGG) in the right insular–temporal site (patient 1), a metastatic medulloblastoma (patient 2), a right intraventricular occipital–parietal choroid plexus carcinoma (patient 3), and a diffuse HGG spreading from the left nuclear thalamus (patient 4). The presenting symptoms were related to the tumor location: gaze deviation to the left and left upper limb seizure in patient 1, gait instability in patient 2, vomiting and drowsiness in patient 3, and right-sided hyposthenia in patient 4. At diagnosis, all patients were subjected to partial neurosurgical removal and induction therapy according to the Italian program for malignant CNS tumors under 3 years schedule. Response was determined with magnetic resonance imaging (MRI) according to the RECIST criteria (9). At post-induction re-evaluation, in patient 1, progressive disease could not be excluded, while the other patients presented stable disease. Two patients (patients 3 and 4) received second-look surgery after induction to achieve better disease control. At the end of the induction treatment, all patients developed reversible comorbidities, such as reduced feeding and asthenia, which are typical of oncological patients, but are more pronounced in such infant pediatric age.

The patients, diagnoses, and treatments before GemOx are summarized in **Table 1**.

**Table 1:** Patients, diagnosis, and treatments before gemcitabine–oxaliplatin (GemOx).

Patient no.	Sex	Histology	Age at diagnosis (months)	Therapies before GemOx
1	M	HGG	8	NS + IC AIEOP SNC INFANT
2	M	MM	28	NS + IC AIEOP SNC INFANT
3	F	CPC	14	NS + IC AIEOP SNC INFANT + second-look NS
4	F	Diffuse HGG	24	NS + IC AIEOP SNC INFANT + second-look NS

*M*, male; *F*, female; *HGG*, high-grade glioma; *MM*, metastatic medulloblastoma; *CPC*, choroid plexus carcinoma; *NS*, neurosurgery; *IC*, induction chemotherapy.

To delay HDC for uncertain response to the induction chemotherapy needing re-evaluation, to consider the possibility of a second-look neurosurgery before intensified regimens, and to allow infant patients to adequately recover from previous treatments in view of intensive therapy, given also the extremely young age, between May 2017 and May 2023, the included patients received GemOx cycles as a bridge therapy to HDC. This combination therapy was chosen based on previous promising data in adult advanced solid tumors (1) and in children with relapsed or refractory solid tumors, inclusive of medulloblastoma and other CNS tumors (4), as well as in children affected by mature B-cell NHL who are unfit for intensive chemotherapy (6).

Gemcitabine (as a 100-min infusion, 1,000 mg/m^2^ or 33.3 mg/kg in the case of weight <10 kg) and oxaliplatin (as a 2-h infusion, 100 mg/m^2^ or 3.3 mg/kg in the case of weight <10 kg) were administered on day 1 of each cycle, planned every 21–28 days, dependent on blood count recovery (absolute neutrophil count ≥1,000/μl and platelets ≥100,000/μl) and permissive clinical/performance status. Toxicities were graded according to the Common Terminology Criteria for Adverse Events (CTCAE), version 5.0.

## 3 Results

A total of 14 GemOx courses were administered. The median number of GemOx cycles per patient was 3.5 (range, one to six courses). The courses were given in a day hospital regimen. The total number of administered courses was established based on the purpose and the achievement of the desired clinical status and stable disease response.

At MRI disease assessment, maximum every four cycles, all patients showed optimal disease control (three stable disease and one partial response). After the GemOx cycles, all patients presented good clinical conditions, with good nutritional status, and were recovering from reversible comorbidities and the health decline intrinsically due to the induction chemotherapy. Considering the achieved optimal condition, one patient (patient 2) was also subjected to further surgery. All four patients were safely exposed to HDC (one to two courses) with subsequent autologous stem cell transplant, without complications (two HDC courses in patients 1 and 2 and one HDC course in patients 3 and 4). One or two courses of HDC were administered according to the clinical status, age of the patient, histology, and the attainment of pretreatment goals based on primary tumor extension.

At the end of HDC, three patients presented disease stability. However, the disease was progressive in one patient (patient 2); therefore, a radiotherapy course was delivered considering the achievement of permissive age. Patient 3 progressed 19 months after the end of GemOx, requiring further surgery (gross total resection, GTR) and radiotherapy.

**Figure 1:**
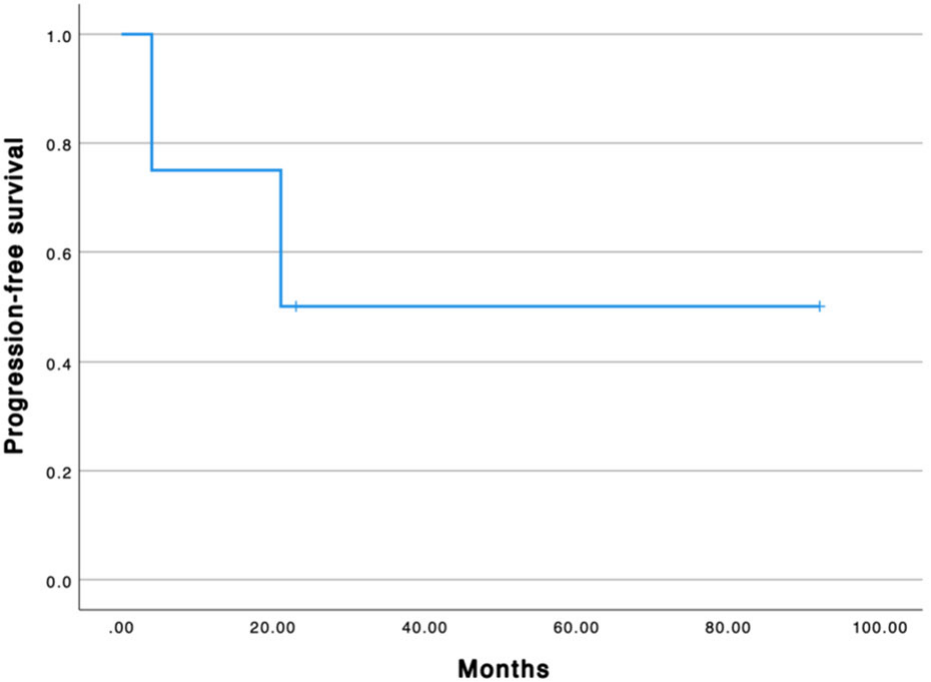
Progression free survival curve.

The median progression-free survival from the first GemOx is 22 months (**Figure 1**). All patients are alive. The median overall survival from the first GemOx is 65 months.

GemOx was well tolerated overall, with the most common toxicities being hematologic (**Table 2**). Only one patient had grade 4 thrombocytopenia during therapy, which required dose reduction to 75% of the total dose. The same patient presented febrile neutropenia CTCAE 3. No infusion reactions or other acute adverse events were reported. No granulocyte colony-stimulating factor was required among courses.

**Table 2:** Gemcitabine–oxaliplatin (GemOx) response and toxicity and post-GemOx treatments.

Patient no.	GemOx course	GemOx interval (days)	GemOx response	Toxicity	Treatment after GemOx	Progressive disease (months after GemOx)	Other therapy
1	3	21	SD	Hb CTCAE1 Seizure CTCAE1	HDC (two courses)	NO	
2	1	–	SD	–	Surgery + HDC (two courses)	YES (4)	RT
3	4	28	PR	–	HDC (one course)	YES (19)	NS+RT
4	6	21	SD	Hb CTCAE 1 Plt CTCAE 4 Febrile neutropenia CTCAE 3	HDC (one course)	NO	

*SD*, stable disease; *PR*, partial response; *Hb*, hemoglobin; *Plt*, platelets; *CTCAE*, Common Terminology Criteria for Adverse Events; *HDC*, high-dose chemotherapy; *NS*, neurosurgery; *RT*, radiotherapy.

The GemOx response and toxicity and the post-GemOx treatments are reported in **Table 2**.

## 4 Discussion

In this infant malignant brain tumor cohort, the GemOx combination therapy represented a safe and encouraging strategy as a bridge therapy to HDC and autologous transplantation. The bridge time before intensive therapy was extremely useful for the achievement of clinical permissiveness after previous treatments and for better disease status and responsiveness evaluation. To date, there are no alternative strategies for infant patients with brain tumors temporarily not suitable to receive HDC.

The GemOx combination therapy was chosen based on previously reported promising data. It is known that the GemOx combination is active and well tolerated in advanced biliary tract adenocarcinoma (10), representing a standard first-line treatment (11). In this setting, further studies are emerging on its combination with immune checkpoint inhibitors (12) or with anti-programmed cell death ligand 1/vascular endothelial growth factor inhibitors (13).

GemOx has been studied as a first-line therapy for advanced pancreatic cancer (14), showing evidence of activity in a phase II trial (15). In recent years, in this context, it has been combined with encouraging treatments, such as high-intensity focused ultrasound (HIFU) ablation, showing clinical benefits (16).

GemOx has also been considered in other solid cancers. In 2006, Kakolyris et al. evaluated the activity and tolerance of this regimen in pretreated patients with advanced non-small cell lung cancer (NSCLC), showing relative activity and good tolerance (17). In a phase III randomized trial, GemOx was also evaluated against carboplatin–paclitaxel as a first-line therapy in this type of cancer (18). In 2009, Ray-Coquard et al. reported on the activity of GemOx in advanced ovarian carcinoma in early progression resistant to taxane–platinum treatment (19). In 2013, Vici et al. also examined the efficacy and safety of GemOx in recurrent, platinum-resistant ovarian cancer in a multicenter phase II clinical trial, showing encouraging activity and manageable toxicity (20). In 2007, Caruba et al. investigated the efficacy and safety of GemOx in patients with metastatic breast cancer heavily treated with anthracycline and taxane (21), while in 2017 Rizzo et al. reported GemOx as an active regimen in the treatment of luminal and human epidermal growth factor receptor 2-positive metastatic breast cancer (22). GemOx has also been proposed in urogenital tumors (23). A phase II study on GemOx was conducted in cisplatin-”unfit” patients with stage IV transitional cell bladder cancer (23). Pectasides et al. performed a phase II study evaluating GemOx in patients with cisplatin-refractory germ cell tumors, showing an encouraging 14% long-term disease-free status (24). GemOx was also assessed in recurrent nasopharyngeal carcinoma (25). In a phase II trial involving previously untreated patients with advanced hepatocellular carcinoma (HCC), the GemOx regimen appeared to be well tolerated and active (26). A large retrospective multicenter study on advanced HCC confirmed that GemOx is effective with manageable toxicity, permitting potentially curative treatment that was not initially feasible in a significant proportion of patients (27).

In view of the encouraging reported data on the adult population, GemOx has been evaluated in children with relapsed or refractory solid tumors, inclusive of medulloblastoma and other CNS tumors (4). In a biweekly schedule, an acceptable safety profile was reported, but with limited activity (4).

With regard to the hematological field, it has shown high efficacy with a low toxicity profile in elderly patients with relapsed and refractory diffuse large B-cell lymphoma (28), and it has been studied as an additional option in patients with recurrent/refractory primary CNS lymphomas (29). It was also evaluated in combination with rituximab as a viable treatment option for children with NHL who are unfit for intensive chemotherapy (6). Considering other GemOx add-on therapies, in The Lancet, the STARGLO trial by Abramson et al. introduced glofitamab (Glofit), a CD20 × CD3 bispecific antibody, combined with GemOx as a promising alternative for transplant-ineligible patients, meeting its primary endpoint (30). Otham et al. evaluated the safety and efficacy of atezolizumab plus rituximab and GemOx (R-GemOx+Atezo) in relapsed/refractory diffuse large B-cell lymphoma transformed from indolent B-cell lymphomas, demonstrating tolerance and promising preliminary efficacy (31). Moreover, in a multicenter, single-arm, phase 2 trial, Tian et al. assessed the safety and activity of another programmed cell death protein 1 inhibitor plus P-GemOx (pegaspargase, gemcitabine, and oxaliplatin) in the first-line setting for advanced extranodal natural killer/T-cell lymphoma, also showing safeness and activity in this case (32). For solid tumors, Dong et al. explored the performance of GemOx plus immunotherapy and next-generation sequencing (NGS)-guided targeted therapy for local advanced or metastatic biliary tract cancer, showing promising data (33). A different route of administration for GemOx has also been proposed: GemOx hepatic arterial infusion chemotherapy plus systemic chemotherapy in combination with an immune checkpoint inhibitor has been recently administered in patients with large unresectable intrahepatic cholangiocarcinoma (34). The combination of GemOx and immunotherapy was assessed as a first-line therapy for advanced intrahepatic cholangiocarcinoma, opening the landscape for a phase III, multicenter, double-blind, randomized study to validate the findings (35). A few studies have explored the combination of GemOx and bevacizumab. In 2023, Wang et al. investigated the efficacy and safety of atezolizumab, bevacizumab, and GemOx in advanced biliary tract cancer, exploring the potential biomarkers related to the response (13). In 2018, Bréchon et al. assessed and compared the efficacy of GemOx plus bevacizumab and GemOx alone in metastatic carcinoma of the biliary tract, concluding with an increase in progression-free survival and manageable toxicity with the combination therapy (36). Antiangiogenic therapy has been the most investigated strategy for glioblastoma in the last decade (37), and bevacizumab-containing regimens have shown promising activity in relapsed/refractory brain tumors (38). In our opinion, this could be an inspiring GemOx add-on avenue for research in the field of brain cancer.

There are different treatment schedules, and not one is universally established, the most common being gemcitabine over 30 or 100 min on days 1 and 8 and oxaliplatin as a 2-h infusion on day 1 or day 8 of a 21-day cycle or biweekly schedules with gemcitabine on day 1 and oxaliplatin on day 1 or day 2 of a 14-day cycle (4). In our cohort, as GemOx was used as a bridge capable of maintaining disease control rather than a salvage treatment, and taking the frail age of the patients into consideration, we decided to prolong the inter-cycle interval, administering GemOx on day 1 of a 21- to 28-day cycle.

Myelosuppression, asthenia, and nausea/vomiting have been reported as the major toxicities of gemcitabine (1). On the other hand, cumulative peripheral neurotoxicity is the main side effect of oxaliplatin (1). Demols et al. described that grade III/IV non-neurologic toxicities occurred in 36.3% of GemOx-treated adult patients, while grade I, II, and III neuropathy occurred in 51%, 9%, and 12% patients, respectively (14). Raspagliesi et al. reported thrombocytopenia as the dose-limiting toxicity in patients who received GemOx as a second-line treatment for refractory ovarian cancer (39). However, it must be considered that patients treated with GemOx are usually heavily pretreated with chemotherapy; therefore, the add-on toxicity might play a role. In our cohort, one patient experienced grade 4 thrombocytopenia during GemOx therapy, which required dose reduction. No neurotoxicity was reported during the follow-up.

There are a few therapeutic options other than intensive chemotherapy up to HDC that are possible in children under 3 years with cerebral tumors who should not be exposed to radiotherapy, being more vulnerable to hypothalamic–pituitary dysfunction (40) and to the risk of cognitive sequelae that impact long-term quality of life (41). To date, data on HDC unfitness have been limited.

Moreover, neoadjuvant chemotherapy may reduce the tumor vascularity and volume (42), facilitating maximal tumor resection (43): a GTR of pediatric brain tumors is one of the most important predictors of outcome (42). Second-look surgery appears to be feasible, safe, and effective with regard to the volumetric outcome parameters (44). Based on individual cases, GemOx bridge treatment may also permit a more accurate evaluation of the possibility of reintervention to safely achieve maximal tumor resection.

In conclusion, combination treatment with GemOx should be a viable option strategy in partially removed infant brain tumors in the case of temporary unsuitability for intensified treatments or while waiting for evolution verification, especially in radiotherapy delaying programs. In our cohort, the therapy was overall safe and well tolerated, except for the grade 4 hematological toxicity in only one patient. Although this is a small case series, based on our experience, the GemOx approach should result incisive in the therapeutic or palliative choice for extremely young patients with aggressive brain tumors.

## Data Availability

The original contributions presented in the study are included in the article/supplementary material. Further inquiries can be directed to the corresponding author.
